# *Notes from the Field:* Fatal *Acanthamoeba* Encephalitis in a Patient Who Regularly Used Tap Water in an Electronic Nasal Irrigation Device and a Continuous Positive Airway Pressure Machine at Home — New Mexico, 2023

**DOI:** 10.15585/mmwr.mm7410a4

**Published:** 2025-03-27

**Authors:** Julia C. Haston, Ibne K. Ali, Shantanu Roy, Alexis Roundtree, Jessica Hofstetter, Savannah Pierson, Emily Helmrich, Paul Torres, Kodi Lockey, Roosecelis B. Martines, Mia Mattioli

**Affiliations:** ^1^Division of Foodborne, Waterborne, and Environmental Diseases, National Center for Emerging and Zoonotic Infectious Diseases, CDC; ^2^New Mexico Department of Health, Albuquerque, New Mexico; ^3^Office of the Medical Investigator, University of New Mexico, Albuquerque, New Mexico; ^4^Division of High-Consequence Pathogens and Pathology, National Center for Emerging and Zoonotic Infectious Diseases, CDC.

**Summary**

**What is already known about this topic?**

*Acanthamoeba*, a free-living ameba, can cause encephalitis and disseminated disease that are nearly always fatal. Immunocompromised persons are at highest risk for these infections.

**What is added by this report?**

In November 2023, a patient died from an *Acanthamoeba* infection, likely acquired by using tap water in electronic medical devices. *Acanthamoeba* was detected in the patient’s brain tissue, an electronic nasal irrigator, and a continuous positive airway pressure (CPAP) machine; all strains were of the same genotype.

**What are the implications for public health practice?**

Patients should always follow manufacturer instructions regarding the type of water to use and recommended cleaning practices for electronic medical devices such as CPAP machines. Distilled, sterile, or boiled and cooled tap water can be used in nasal irrigation devices.

*Acanthamoeba* is a genus of free-living ameba that can cause severe disease of the brain, eyes, sinuses, skin, and other organs, particularly among immunocompromised persons. Approximately three to 12 persons are infected with nonkeratitis *Acanthamoeba* infections in the United States annually, and a majority die (*1*). Because of the unknown incubation period of *Acanthamoeba* spp., which might be weeks or months, and its ubiquity in the environment, the source of exposure is typically unknown. In a case series of ten immunocompromised patients with nonkeratitis *Acanthamoeba* infection, all reported performing nasal irrigation before becoming ill, many using tap water, but no confirmation of this exposure route through environmental testing was reported (*2*). This report confirms the link between intranasal exposure to contaminated tap water and the development of *Acanthamoeba* granulomatous amebic encephalitis in an older patient and highlights the risk associated with using tap water in electronic medical devices.

## Investigation and Outcomes

On November 15, 2023, CDC was notified of a patient aged 66 years who had died approximately 3 weeks after being hospitalized for altered mental status and weakness. Symptoms progressed to include seizures, fever, and respiratory and gastrointestinal complications. Brain lesions were noted on magnetic resonance imaging and, at autopsy, histopathologic evidence of granulomatous amebic encephalitis was identified. The patient had reported no recent recreational water exposure but regularly used tap water in an electronic nasal irrigation device and a continuous positive airway pressure (CPAP) machine at home. Information about how these devices were cleaned was not available. The patient had a history of diabetes mellitus, obstructive sleep apnea, alcohol use disorder, and ulcerative colitis requiring a total colectomy.

On January 4, 2024, CDC received patient brain specimens for testing through coordination with the New Mexico Department of Health. The diagnosis of granulomatous amebic encephalitis caused by *Acanthamoeba* (Figure) was confirmed using an *Acanthamoeba* species immunohistochemical assay and polymerase chain reaction (PCR) (*3*). *Acanthamoeba* was also detected by culture in the electronic nasal irrigation device and in the drained water receptacle from the patient’s CPAP machine, followed by real-time PCR confirmation on February 5* (*3*). All detected *Acanthamoeba* strains belonged to the T4 genotype, which is the most common genotype detected among encephalitis cases^†^ (*4*,*5*). This activity was reviewed by CDC, deemed not research, and was conducted consistent with applicable federal law and CDC policy.^§^

**FIGURE:**
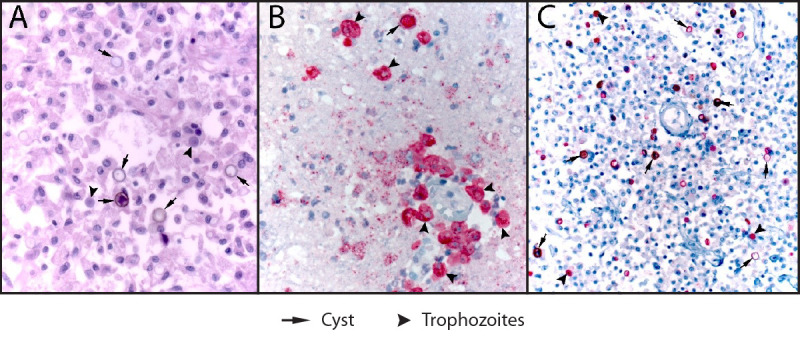
Histopathologic findings in a fatal case of granulomatous amebic encephalitis caused by *Acanthamoeba* T4 genotype* Photos/Infectious Disease Pathology Branch, CDC * Extensive granulomatous inflammation and necrosis surrounding cysts and trophozoites of *Acanthamoeba* species in brain tissue (A). An *Acanthamoeba* species immunohistochemical assay highlighted amebic antigens in cysts and trophozoites located in subacute perivascular microabscesses (B) and areas of chronic granulomatous inflammation (C).

## Preliminary Conclusions and Actions

Prevention of *Acanthamoeba* infections has been challenging because of lack of information about risk behaviors and transmission of this environmentally ubiquitous pathogen. Although nearly all cases occur among immunocompromised persons, the route of transmission is unknown for a majority of cases. This case investigation confirms that intranasal exposure to tap water can cause *Acanthamoeba* infection. Inadequate cleaning and drying of nasal irrigation devices and medical devices might have been contributing factors in this case, given that some of these devices have parts that are difficult to access for proper cleaning and drying. Although more work is needed to elucidate whether the risk for *Acanthamoeba* infection might be increased by inadequate cleaning practices, all persons who use nasal irrigation devices or electronic medical devices should follow cleaning guidance provided by the manufacturer. Health care providers should consider counseling patients about *Acanthamoeba* infections and encourage the use of distilled, sterile, or boiled and cooled tap water when performing nasal irrigation and adherence to manufacturer recommendations when using electronic medical devices such as CPAP machines.^¶^^,^**

CDC offers a 24-hour, 7 days-a-week free-living ameba clinical consultation service to provide diagnostic and treatment advice to health care providers.^††^ Clinicians are encouraged to report cases of *Acanthamoeba* infection to local or state public health officials. CDC recommends that public health officials report cases to CDC to enhance ongoing surveillance activities.

## Footnotes

* *Acanthamoeba* spp. testing included rinsing objects three times with a total of 175 ml William Balamuth saline/0.01% Tween 80 solution. The rinse solution was then pelleted by centrifugation at 1500 x *g* for 15 minutes at 77°F (25°C), followed by culture for amebas on nonnutrient agar with *Escherichia coli* lawn at 86°F (30°C) for ≤14 days. Specimens with observed ameba via microscopy were scraped for genomic testing using the ZymoBIOMICS DNA/RNA Miniprep kit for nucleic acid extraction and real-time PCR.

† Sanger amplicon sequencing of a region of the 18S rRNA gene was conducted to determine genetic relatedness of the isolate genotypes to one another.

§ 45 C.F.R. part 46, 21 C.F.R. part 56; 42 U.S.C. Sect. 241(d); 5 U.S.C. Sect. 552a; 44 U.S.C. Sect. 3501 et seq.

¶
https://www.cdc.gov/naegleria/prevention/sinus-rinsing.html

**
https://www.cdc.gov/drinking-water/prevention/preventing-waterborne-germs-at-home.html

†† The CDC Free-Living Ameba Clinical Consultation service can be reached by calling the CDC Emergency Operations Center at 770-488-7100.
